# Mitochondrial genome of *Aphis gossypii* Glover cucumber biotype (Hemiptera: Aphididae)

**DOI:** 10.1080/23802359.2021.1888328

**Published:** 2021-03-16

**Authors:** Ruichang Niu, Xueke Gao, Junyu Luo, Li Wang, Kaixin Zhang, Dongyang Li, Jichao Ji, Jinjie Cui, Xiangzhen Zhu, Shuai Zhang

**Affiliations:** a Institute of Cotton Research of Chinese Academy of Agricultural Sciences, State Key Laboratory of Cotton Biology, Anyang, Henan, China; b School of Horticulture and Plant Protection, Yangzhou University, Yangzhou, Jiangsu, China

**Keywords:** Cotton-melon aphid, mitogenome, cucumber biotype, cotton biotype, phylogenetic analysis

## Abstract

The complete mitochondrial genome of *Aphis gossypii* Glover cucumber biotype was sequenced using traditional PCR amplification coupled with Sanger sequencing. The genome is 15,870 bp long, with 83.7% AT content (MW048625). The genome encodes 37 typical mitochondrial genes, including 13 protein-coding genes, 2 ribosomal RNA genes, 22 transfer RNAs, a repeat region of 784 bp, and a control region of 627 bp. The base composition of the genome is A (45.4%), T (38.3%), C (10.5%), and G (5.8%). An analysis of two biotypes *A. gossypii* mitogenomes identified 77 single nucleotide polymorphisms and 1 insertion and deletion.

The *Aphis gossypii* Glover, 1877 is one of the most widely encountered insect pest in the agricultural flora and fauna. It often afflicts plants such as melon, marrow, zucchini, watermelon (*Cucurbitaceae*), cotton, okra, ornamental hibiscus from *Malvaceae* and potato, chili pepper, sweet pepper, eggplant from *Solanaceae* (Carletto et al. 2009). *Aphis gossypii* feeds on plant sap, which can eventually kill the plant and can also transmit several viruses (Webster et al. 2018).

To date, different host specialization types of aphids have evolved from which cucumber and cotton biotypes are prominently distributed (Zhang et al. 2018). Significant difference was recorded among different adaptive hosts of aphids. The biotype belonging to cotton cannot subsist in cucumber and similarly cucumber biotype cannot flourish normally in cotton crop (Wang et al. 2015). We have previously reported the mitochondrial genome of *A. gossypii* cotton biotype (Zhang et al. 2016). To investigate intra-specific variations and phylogenetic relationship of *A. gossypii* cucumber biotype, we determined the complete sequence of the mitochondrial genome of cucumber biotype. The extracted DNA of a single aphid (collected on cucumber, 36°3′43″N, 114°20′54″E) was used as a template for PCR. After sampling, the specimen (N201906) was stored in the State Key Laboratory of Cotton Biology, Institute of Cotton Research of Chinese Academy of Agricultural Sciences, Anyang, Henan Province, China. The cucumber biotype of *A. gossypii* was initially identified according to earlier reported studies (Wang et al. 2015). Primers were designed according to cotton biotype mitochondrial genome sequence (Supplementary Table 1), and PCR products were sequenced in both directions. Then, we assembled the complete mitochondrial genome of *A. gossypii* cucumber biotype using Vector NTI 11.5.1. The online software MITOS2 （http://mitos2.bioinf.uni-leipzig.de/index.py） was used to annotate the splicing sequence of the mitochondrial genome of cucumber biotype (Torres et al. 2019) with *A. gossypii* mitochondrial genome as a reference (KJ669654; Zhang et al. 2016).

The mitochondrial genome of cucumber biotype is a circular molecule with a length of 15870 bp, which is 1 bp longer than the cotton biotype. The mitochondrial genome contains 13 protein coding genes, 2 ribosomal RNA genes, 22 tRNA genes, a control region of 627 bp, and a repeat region of 784 bp (Additional Table 2). The repeat region includes three 196-bp tandem repeats (near tRA^Glu^) and one 189-bp copy of the anterior portion of the repeat unit. In general, the content of A + T in the mitochondrial genome of *A. gossypii* cucumber biotype was 83.7% (A = 45.4%; T = 38.3%; C = 10.5%; G = 5.8%). The A + T content was identical to that of cotton biotype (Zhang et al. 2016). Comparing the two biotypes of *A. gossypii* mitogenomes, 77 single nucleotide polymorphisms (SNPs) and 1 insertion and deletion (INDEL) were recognized. The 77 single nucleotide polymorphisms comprised of a molecular marker with 5 single nucleotide polymorphisms, which were stable and can be used to identify *A. gossypii* biotypes. These five poly nucleotides are located in the region of Cytb gene. The nucleotides present at the five polymorphic positions were T, A, A, T, and T in the fragment of cotton biotype and C, G, G, C, and C in the fragment of cucumber biotype.

To confirm the phylogenetic position of *A. gossypii* cucumber biotype, a total of 39 complete mitochondrial genomes of Aphidoidea superfamily were obtained from GenBank, and *Bemisia tabaci* was used as outgroup. Multiple sequence alignment was conducted by MAFFT 7.388 (Katoh and Standley, 2013). Phylogenetic analysis was conducted based on maximum likelihood (ML) analyses implemented in IQ-TREE 1.5.5 (Nguyen et al. 2015) under the GTR + F + I + G4 nucleotide substitution model, which was selected by ModelFinder (Kalyaanamoorthy et al. 2017). Support for the inferred ML tree was inferred by bootstrap-ping with 1000 replicates. *Aphis gossypii* cucumber biotype is clustered with cotton biotype, as expected, and all mitochondrial genomes of Aphis genus form one clade, presenting monophyletic manner (Figure 1). In addition, the Aphididae family including *A. gossypii* cucumber biotype represented a clear monophyletic relationship (Figure 1), whereas the Aleyrodidae family are paraphyletic. In conclusion, the mitochondrial genome will be helpful to understand the phylogenetic relationship of the Aphidoidea clade.

**Figure 1.**
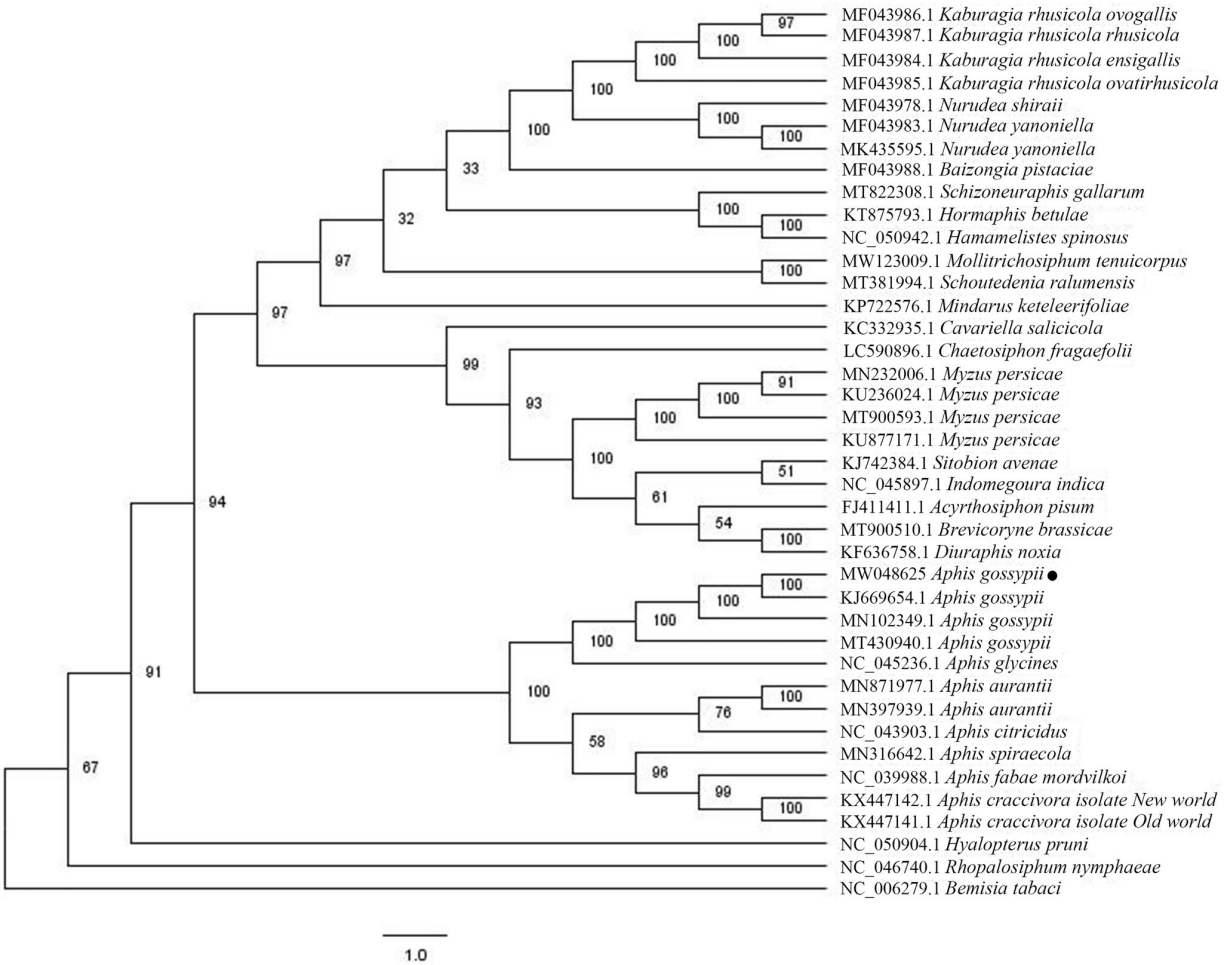
Maximum-likelihood phylogenetic tree inferred from 40 mitochondrial genomes. The number on each node indicates bootstrap support value. Our *A. gossypii* mitochondrial genome is highlighted using black dot.

## Data Availability

The data that support the findings of this study are openly available in Additional tables and NCBI GenBank database at https://www.ncbi.nlm.nih.gov with the accession numbers MW048625 which permits unrestricted use, distribution, and reproduction in any medium, provided the original work is properly cited.

## References

[CIT0001] Carletto J, Lombaert E, Chavigny P, Brevault T, Lapchin L, Vanlerberghe-Masutti F. 2009. Ecological specialization of the aphid *Aphis gossypii* Glover on cultivated host plants. Mol Ecol. 18(10):2198–2212. 19635073 10.1111/j.1365-294X.2009.04190.x

[CIT0002] Kalyaanamoorthy S, Minh BQ, Wong TKF, von Haeseler A, Jermiin LS. 2017. ModelFinder: fast model selection for accurate phylogenetic estimates. Nat Methods. 14(6):587–589. 28481363 10.1038/nmeth.4285 PMC5453245

[CIT0003] Katoh K, Standley DM. 2013. MAFFT multiple sequence alignment software version 7: improvements in performance and usability. Mol Biol Evol. 30(4):772–780. 23329690 10.1093/molbev/mst010 PMC3603318

[CIT0004] Nguyen L-T, Schmidt HA, Von Haeseler A, Minh BQ. 2015. IQ-TREE: a fast and effective stochastic algorithm for estimating maximum-likelihood phylogenies. Mol Biol Evol. 32(1):268–274. 25371430 10.1093/molbev/msu300 PMC4271533

[CIT0005] Torres L, Welch AJ, Zanchetta C, Chesser RT, Manno M, Donnadieu C, Bretagnolle V, Pante E. 2019. Evidence for a duplicated mitochondrial region in Audubon's shearwater based on MinION sequencing. Mitochondrial DNA Part A. 30(2):256–263. 10.1080/24701394.2018.1484116 30043666

[CIT0006] Wang L, Zhang S, Luo JY, Lv LM, Wang CY, Cui JJ. 2015. Host biotypes of cotton aphid *Aphis gossypii* Glover and preliminary analysis of the formation mechanism in Anyang region of China. Cotton Sci. 27:372–378.

[CIT0007] Webster CG, Pichon E, van Munster M, Monsion B, Deshoux M, Gargani D, Calevro F, Jimenez J, Moreno A, Krenz B, et al. 2018. Identification of plant virus receptor candidates in the stylets of their aphid vectors. J Virol. 92(14):e00432–18. 29769332 10.1128/JVI.00432-18 PMC6026765

[CIT0008] Zhang S, Luo JY, Wang CY, Lv LM, Li CH, Jiang WL, Cui JJ, Rajput LB. 2016. Complete mitochondrial genome of *Aphis gossypii* Glover (Hemiptera: Aphididae). Mitochondrial DNA Part A. 27(2):854–855. 10.3109/19401736.2014.919474 24865902

[CIT0009] Zhang S, Luo J-y, Wang L, Wang C-y, Lü L-m, Zhang L-j, Zhu X-z, Cui J-j. 2018. The biotypes and host shifts of cotton-melon aphids *Aphis gossypii* in Northern China. J Integrat Agri. 17(9):2066–2073.

